# AdDRESSing T-cell responses to antituberculous drugs

**DOI:** 10.1111/bjd.15167

**Published:** 2017-02

**Authors:** R. Pavlos, A. Redwood, E. Phillips

**Affiliations:** 1 Institute for Immunology and Infectious Diseases, Murdoch University, Murdoch, Australia; 2 Departments of Medicine, Pathology, Microbiology, Immunology and Pharmacology, Vanderbilt University Medical Center, Nashville, TN, U.S.A

In this issue of the *BJD*, Ye *et al.*^1^ present data on T-cell-specific responses in patients with antituberculosis drug (ATD)-induced maculopapular exanthema (MPE) and drug reaction with eosinophilia and systemic symptoms (DRESS). Understanding these responses helps further our knowledge of the immunopathogenesis of antituberculous hypersensitivity, and ideally it would be translated into diagnostic tests that improve ATD drug safety and guide therapy. Isoniazid, rifampicin, pyrazinamide and ethambutol are the first-line therapy used in the first 2 months to treat tuberculosis (TB) and multiple medications make the identification of culprit drugs difficult in the clear diagnosis of adverse drug reactions (ADRs). This uncertainty has detrimental effects, including interruption of treatment for prolonged periods, systemic corticosteroid use and alternative treatment with less effective regimens.^2^ To confirm the diagnosis of suspected immunologically-mediated ADRs associated with ATD therapy, a combination of skin tests (prick and intradermal dilutional testing) and patch testing is most often employed in the clinic. However, the specificity and sensitivity of patch testing is dependent on both the host and the offending drug and few validation studies exist. In the cases presented by Ye *et al.*^1^ patch testing is shown to be of little utility in DRESS and is unsuitable for cases of MPE; however, the oral provocation test and lymphocyte transformation test show a stronger correlation and support multiple drug reactivity.

Another confounding factor in ATD-associated ADRs is the human immunodeficiency virus (HIV) comorbidity present in 12% of newly diagnosed patients with TB.^3^ In this subset of HIV–TB coinfected patients, systemic reactions to patch testing with rifampicin, isoniazid, pyrazinamide and ethambutol have been reported to occur in as many as 90% of patients^4^ and the delay in treatment of ATD is detrimental. Multidrug causality in ATD-associated DRESS has also been indicated in HIV-infected patients.^5,6^

The study by Ye *et al.*^1^ provides insight into the mechanism of ATD-associated adverse reactions supporting a role of drug-specific CD4^+^ T cells in the pathogenesis. The authors show that drug-specific CD4^+^ T-cell clones can be generated for both isoniazid and rifampicin in patients with both MPE and DRESS, with two cases showing the presence of specific CD4 T-cell clones for both drugs. Interferon-γ/granzyme B secretion are dose-dependent and drug-specific and require the presence of both soluble drug and antigen-presenting cells. Finally, blocking experiments demonstrate that the CD4^+^ T-cell response is major histocompatibility complex class II restricted. Existing human leucocyte antigen (HLA) data is minimal in relation to anti-TB drugs. There has been an association reported in Korean patients with DRESS for the class I allele HLA-C*04:01,^7^ which extends to the haplotype HLA-A* 11:01-B*15:01-C*04:01. However, these alleles are not reported in the cases presented by Ye *et al.*,^1^ providing evidence that other class II HLA alleles may be important.

Future work is required to confirm multiple drug hypersensitivity in patients on ATD therapy suggested by the data of Ye *et al.*^1^ and the mechanism of HLA–drug–CD4^+^ T-cell interactions and where possible testing strategies including genetic, *in vivo* approaches and *ex vivo* assays to support safe oral challenge should be incorporated into prospective trial designs where standardized phenotyping and testing procedures can be applied. The ability to accurately confirm the causative drug in ATD-induced ADRs will allow safer management and critical continuation of the therapy required for the efficacious treatment of TB, particularly for patients who are HIV-positive.
